# Altered Intrinsic Regional Activity and Interregional Functional Connectivity in Post-stroke Aphasia

**DOI:** 10.1038/srep24803

**Published:** 2016-04-19

**Authors:** Mi Yang, Jiao Li, Yibo Li, Rong Li, Yajing Pang, Dezhong Yao, Wei Liao, Huafu Chen

**Affiliations:** 1 Center for Information in BioMedicine, Key Laboratory for Neuroinformation of Ministry of Education, School of Life Science and Technology, University of Electronic Science and Technology of China, Chengdu 610054, China; 2 Department of Stomatology, the Fourth people’s Hospital of Chengdu, Chengdu 610036, China

## Abstract

Several neuroimaging studies have examined cerebral function in patients who suffer from aphasia, but the mechanism underlying this disorder remains poorly understood. In this study, we examined alterations in the local regional and remote interregional network cerebral functions in aphasia combined with amplitude of low-frequency fluctuations and interregional functional connectivity (FC) using resting-state functional magnetic resonance imaging. A total of 17 post-stroke aphasic patients, all having suffered a stroke in the left hemisphere, as well as 20 age- and sex-matched healthy controls, were enrolled in this study. The aphasic patients showed significantly increased intrinsic regional activity mainly in the contralesional mesial temporal (hippocampus/parahippocampus, [HIP/ParaHIP]) and lateral temporal cortices. In addition, intrinsic regional activity in the contralesional HIP/ParaHIP was negatively correlated with construction score. Aphasic patients showed increased remote interregional FC between the contralesional HIP/ParaHIP and fusiform gyrus, but reduced FC in the ipsilesional occipital and parietal cortices. These findings suggested that the intrinsic regional brain dysfunctions in aphasia were related to interregional functional connectivity. Changes in the intrinsic regional brain activity and associated remote functional connectivity pattern would provide valuable information to enhance the understanding of the pathophysiological mechanisms of aphasia.

Aphasia is one of the most disabling cognitive deficits that follow an acute or chronic stroke condition^1^. Post-stroke aphasia nearly always results from left-hemisphere lesions, thereby leading to substantial functional disability and high psychological distress^2^. Although the fine-grain language architecture of aphasia has been extensively examined, dynamic neurobiological mechanisms underlying post-stroke aphasia remain poorly understood^3^. We investigated the influence of cortical lesions in post-stroke aphasia. We considered not only intrinsic regional activity abnormalities, but also interregional functional connectivity (FC) deficits in the brain.

Brain oscillatory modulations were investigated by blood oxygen level-dependent functional magnetic resonance imaging (fMRI) signals^4^. The amplitude of spontaneous brain oscillations was measured as amplitude of low-frequency fluctuations (ALFF) to investigate the disturbances in the intrinsic regional activity in post-stroke aphasia^5^. Abnormalities in the intrinsic FC of remote brain regions have been previously examined in post-stroke aphasia^5^,^6^. Previous studies have found that that the dominant frontoparietal and default mode networks exhibited impaired remote intrinsic FC^7^,^8^. In addition, other intrinsic FC studies examined the language reorganization in stroke patients with^9^ or without aphasia^10^. These studies have suggested that the intrinsic FC in the language network is related to receptive language outcome. Furthermore, integration in the posterior areas of the default-mode network was improved after intensive therapy, and was concurrent with language improvement^11^. These studies suggested that either regional activity or interregional connectivity was altered in aphasic patients. However, the combination of regional cerebral function and functional integration has not been investigated in post-stroke aphasic patients.

We used two resting-state functional MRI metrics to characterize the changes in the intrinsic regional activity and remote interregional FC in patients with post-stroke aphasia. We predicted that patients would show abnormal regional activity and FC in the frontal, temporal, and parietal cortices. Moreover, we examined the correlations between regional activity values and stroke-related clinical characteristics of the post-stroke aphasic patients.

## Results

### Demographics and clinical characteristics of participants

**Figure 1:**
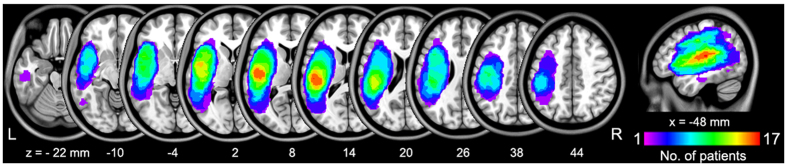
Distribution of the lesion areas for all aphasic patients. The lesion area overlap across patients was rendered on the brain. Colors represent the number of patients with a lesion to a specific voxel. Numbers below each axial map and sagittal map refer to the z-plane and x-plane coordinates of the MNI space, respectively. Letters L and R correspond to the left and right sides of the brain, respectively.

Post-stroke aphasic patients and healthy controls (HC) did not significantly differ in age (two sample t-test, P = 0.98), gender (χ^2^-test, P = 0.90), or years of education (Mann Whitney U-test, P = 0.58) (Table 1). Stroke-related clinical characteristics of the patients were tested using the Aphasia Battery of Chinese (ABC)^12^,^13^. The ABC provides the following information: aphasia quotient (AQ), which includes spontaneous speech, auditory comprehension, repetition, and naming scores; performance quotient (PQ), which includes reading/writing, praxis, and construction (drawing, block design, numerical calculation, and Reven’s colored matrices scores) scores^14^,^15^; and cortical quotient (CQ)^16^ (Table 1). All patients had an ischemic or hemorrhagic stroke in the left hemisphere (Table 2). Lesion overlap images for all aphasic patients are shown in Fig. 1.

### ALFF group differences

**Figure 2:**
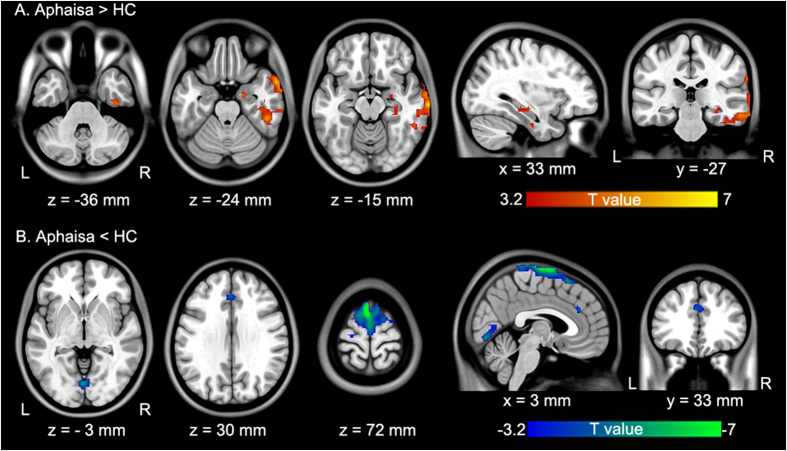
Brain regions showing significant differences in ALFF between aphasic patients and controls. All comparisons were performed using a two-sample t-test (p < 0.05 FDR-corrected and minimum cluster size of 30 voxels). (**A**) Warm colors indicate regions with increased ALFF values in aphasia. (**B**) Cold colors indicate regions with decreased ALFF values in aphasia. Numbers below each sagittal, coronal and axial slice refer to the x-, y-, and z-plane coordinates of the MNI space, respectively. Letters L and R correspond to the left and right sides of the brain, respectively. Further details of these regions are presented in Table 3.

Compared with HCs, post-stroke aphasic patients showed significantly increased ALFF in the contralesional hemisphere, namely, in the hippocampus/parahippocampus (HIP/ParaHip), fusiform gyrus (FFG), inferior temporal gyrus, middle temporal gyrus, and middle temporal pole (false discovery rate (FDR) corrected p < 0.05 and minimum cluster size of 30 voxels). Conversely, aphasic patients indicated significantly reduced ALFF in the ipsilesional hemisphere, particularly, at the superior frontal gyrus, the bilateral precentral gyrus, lingual gyrus, supplementary motor area, and anterior cingulate cortex (FDR corrected p < 0.05 and minimum cluster size of 30 voxels) (Table 3 and Fig. 2).

### Correlations between ALFF and clinical hcharacteristics

**Figure 3:**
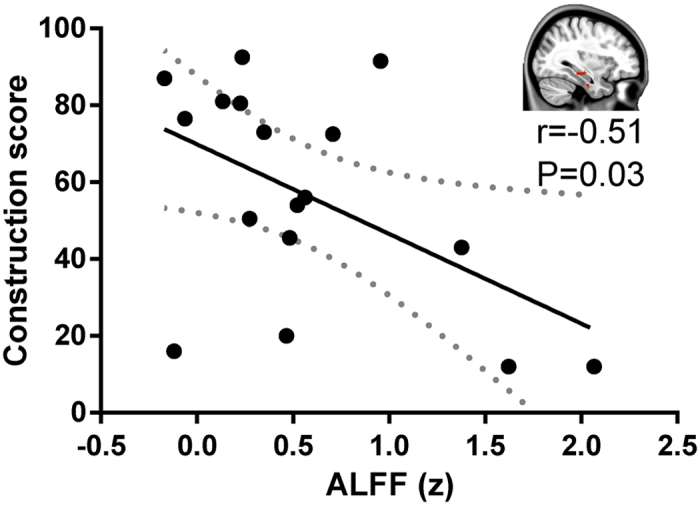
Correlation between ALFF and clinical scores in aphasic patients. ALFF values in the right hippocampus/parahippocampus were negatively correlated with construction scores in the Aphasia Battery of Chinese (r = −0.51, p = 0.03). The solid line and dashed lines represent the best-fit line and 95% confidence interval of Pearson correlation, respectively.

The linear Pearson correlation between altered regional ALFF values and clinical characteristics in aphasic patients was calculated. ALFF in the contralesional HIP/ParaHip was negatively correlated with construction score on the ABC (*r* = −0.51, p = 0.03) (Fig. 3). We found no other significant correlations between the ALFF values in other brain regions and clinical characteristics.

### Altered interregional FC

**Figure 4:**
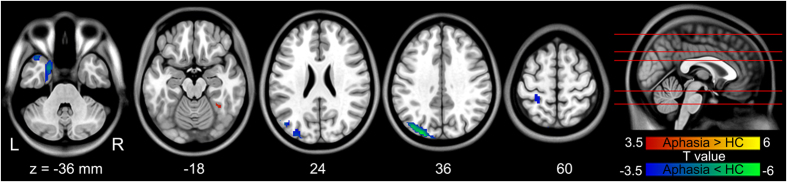
Abnormalities in the seed-based (right hippocampus/parahippocampus) functional connectivity data from aphasic patients. Brain regions showing FC differences between patients with aphasia and controls by two-sample t-test (p < 0.05 FDR-corrected and minimum cluster size of 30 voxels). Warm and cold colors indicate regions with increased and decreased FC values in aphasia, respectively. Numbers below the axial slices refer to the z-plane coordinates of the MNI space, respectively. Letters L and R correspond to the left and right sides of the brain, respectively. Further details of these regions are presented in Table 4.

The contralesional HIP/ParaHip not only show increased ALFF, but it also correlated with construction score on the ABC. Thus, the contralesional HIP/ParaHip was defined as the seed region for subsequent interregional FC analysis. Aphasic patients exhibited increased functional connectivity between the contralesional HIP/ParaHip (seed region) and the contralesional FFG (FDR corrected p < 0.05 and minimum cluster size of 30 voxels). Aphasic patients also showed reduced FC between the contralesional HIP/ParaHip and the ipsilesional middle occipital gyrus, paracentral lobule, postcentral gyrus, and middle/superior temporal pole (FDR corrected p < 0.05 and minimum cluster size of 30 voxels) (Table 4 and Fig. 4).

## Discussion

In this study, we combined ALFF and FC analyses of fMRI data to explore disrupted intrinsic regional activity and interregional functional connectivity in post-stroke aphasic patients. Aphasic patients exhibited significantly increased ALFF values in the contralesional mesial temporal (HIP/ParaHip) and lateral temporal cortices; these patients showed reduced ALFF in the lingual gyrus and frontal cortices. In addition, ALFF in the contralesional HIP/ParaHip was negatively correlated with construction score on the ABC in aphasic patients. Furthermore, aphasic patients showed increased remote interregional FC between the contralesional HIP/ParaHIP and FFG, whereas reduced FC was found between the contralesional HIP/ParaHIP and the ipsilesional occipital and parietal cortices. These findings demonstrate that intrinsic regional brain dysfunction was related to specific network interactions in aphasic patients.

The HIP/ParaHip is thought to be involved in the memory circuit, which is correlated with more severe dementia in the semantic variant of primary progressive aphasia^17^,^18^. In addition, the contralesional parahippocampal activity increased from pre- to post-training, and was correlated with language recovery in chronic aphasia^19^; such as correlation suggested that the contralesional ParaHip may mediate the functional recruitment of the right-sided homologue language regions^20^. In addition, the ALFF in the contralesional HIP/ParaHip was negatively correlated with construction score on the ABC in aphasic patients. Construction ability is characterized by building, copying, and drawing objects^21^. This ability is deficient in patients with left- or right-unilateral stroke lesions^22^. Our correlation analysis suggests that high construction deficit is associated with high intrinsic regional brain activity in the contralesional HIP/ParaHip. The hippocampus is associated with not only episodic memory and spatial navigation, but also scene construction which refers to the ability to describe spatially coherent scenes^23^,^24^. Furthermore, the patients with hippocampal lesions were impaired at constructing scenes^25^. In the present work, the aphasic patients obtained lower construction scores when they constructed various static scenes as fragmented and lacking spatial coherence. Thus, we suggest that the contralesional HIP is predictive of the construction ability in aphasic patients.

Increased ALFF was also observed in the contralesional lateral temporal (such as inferior/middle temporal gyrus, and middle temporal pole) and fusiform gyrus. A previous PET study found distinct contributions from the bilateral inferior temporal poles and the contralesional anterior fusiform gyrus to the semantic processing of speech^26^. A task-related fMRI study of written word and picture semantic processing has found that semantic judgements induced bilateral brain activation in the posterior and anterior temporal middle lobes^27^. Furthermore, the authors found that compared with HCs, aphasic patients displayed an “over-activation” of the bilateral middle temporal lobes while performing semantic judgment tasks^27^. Thus, the temporal lobes are crucial for multimodal semantic processing. Increased intrinsic regional brain activity in the contralesional lateral temporal cortices may be indicative of a compensatory mechanism for semantic processing in aphasia^28^, but such an assertion must be confirmed by a longitudinal evaluation^29^.

Aphasic patients exhibited decreased ALFF values mainly in the ipsilesional frontal cortices. Many functional neuroimaging studies have reported that frontal areas, such as the dorsolateral prefrontal cortex and supplementary motor area, were related with language comprehension and expression, whereas certain frontal regions were not directly included in language but advance comprehension by working memory^30^. Additionally, the precentral gyrus and supplementary motor area are motor speech regions that are influenced in nonfluent variants of primary progressive aphasia. These regions indicate diagnostic potential in aphasic patients^31^, and activity in these frontal regions may be used to predict response of these patients.

Decreased ALFF was also observed in the contralesional lingual gyrus. The lingual gyrus is associated with language and semantic processing^32^, which is considered an essential element of human language^33^. A previous task-related fMRI study demonstrated that the bilateral lingual gyrus is activated in semantic and visual lexical decision and silent reading tasks^34^. Furthermore, the patients with aphasia, as well as the healthy controls, showed right-hemispheric brain activation in the lingual gyrus during word-stem completion task^35^; such a result suggests that right-hemispheric activation indicates the patients’ potential for further language improvement. The present findings suggest that lingual gyrus was closely related to normal integrative functions of language in aphasia not only during task performance, but also at rest.

The intrinsic interregional FC method shows how brain regions work together as networks and how these networks can be enhanced or weakened in aphasia. Aphasic patients also showed decreased FC between the contralesional HIP/ParaHip and ipsilesional parietal lobe. This result was consistent with a previous study in which participants with primary progressive aphasia showed decreased FC in the parietal regions of the left working memory network^36^. Some studies have shown that parietal regions were involved in the language, semantic, and sentence-processing networks. Another task-related study that measured regional brain activities during production and perception in a word-repetition task showed robust responses in the bilateral inferior parietal lobe and premotor cortices^37^.

Aphasic patients also showed decreased FC between the contralesional HIP and ipsilesional middle occipital gyrus, which had a reduced nodal degree and strongly left-lateralized loss of hubs in the semantic variant of aphasic patients^38^. A previous study showed that anomic aphasic patients who had left occipital lesions could not produce normal and detailed descriptions of both abstract and emotional words^39^. Moreover, patients with occipital lesions experienced difficulties in accessing words related to visual modality^39^.

This study presents several methodological limitations. First, the sample size was relatively small, introducing difficulty in obtaining substantial evidence for abnormal local synchronization in aphasia. Second, multiple comparisons for correlations between ALFF and clinical characteristics were not corrected. Third, we ignored the network interaction among the brain regions that showed altered ALFF using region-of-interest based functional connectivity^40^. In future, examining how interaction among regions in a putative language network underlying aphasia is important. Finally, a longitudinal study is needed to examine whether pre-treatment for intrinsic local synchronization may serve as a predictor for prognosis of recovery from aphasia following treatments.

## Conclusion

In summary, we found increased ALFF in the contralesional HIP/ParaHip, which was negatively correlated with construction score on the ABC in aphasic patients. We suggest that intrinsic brain activity in the contralesional HIP/ParaHip predicts the construction ability in aphasia. Aphasic patients exhibited decreased ALFF in the dominant frontal cortices; such a result suggests impaired language and semantic networks. Changes in the intrinsic regional brain activity and associated remote FC network would provide valuable information to enhance understanding of the pathophysiological mechanisms of aphasia.

## Methods

### Subjects

Seventeen aphasic patients (all right-handed, six females and 11 males; age, 53.53 ± 14.06 years) were recruited from admission at Fuzhou Hospital. Patients were recruited according to the following criteria: i) first stroke occurred in the left hemisphere; ii) age of >18 and <85 years; iii) native Chinese speaker; iv) aphasia persistent at day 1 post-stroke; and v) right-handed. Participants were excluded if they had the following: i) any past or current neurological disorders or family history of hereditary neurological disorders; ii) a history of head injury resulting in loss of consciousness; iii) alcohol or substance abuse; iv) claustrophobia; and v) incompatible implants. All patients experienced a single left-hemisphere ischemic (*n* = 15) or hemorrhagic (*n* = 2) stroke (lesion size: 28.85 ± 42.84 cm^3^) and underwent MRI for an average of 9.9 ± 5.4 days after stroke (Table 1). Aphasia persists at this time-point for all patients. All patients in the aphasia and HC groups were right-handed native Chinese speakers.

All patients received a comprehensive evaluation, including medical history and neurological examination, neuropsychological testing, and neuroimaging. Aphasia was diagnosed based on the ABC, which is the Chinese standardized adaptation of the Western Aphasia Battery^12^,^13^. The ABC provides the AQ, PQ, and CQ^16^. AQ reflects a global measure of severity and type of aphasia. AQ (range, 0–100) is derived from linguistic subtests including spontaneous speech, auditory comprehension, repetition, and naming. The normative scores of AQ is 97.11 ± 2.43 (mean ± SD)^41^. The cut-off scores for AQ is 93.25 which based on the receiver operating curve analyses on AQ to differentiate between healthy and aphasic individuals^41^. Anomic (*n* = 9), Broca’s (*n* = 2), and conduction (*n* = 6) aphasia patients were included according to AQ. PQ (range, 0–40) combines scores of reading/writing, praxis, and construction (Drawing, Block design, numerical Calculation, and Reven’s Colored Matrices Score)^14^. CQ is the sum of all subsets based on spontaneous speech score+ auditory comprehension score/10+ repetition score/10+ naming score/10+ reading/writing score/10+ praxis score/6+ construction score/6^14^,^15^. CQ (range, 0–100) is a more general measure of cortical function that provides an overall picture of cognitive status^42^. The normative scores of CQ is 95.57 ± 3.01 (mean ± SD) and the cut-off scores is 90.85^41^.

A total of 20 age-, gender-, and education-matched HCs (all right-handed, eight females and 12 males, 54.05 ± 8.43 years of age) were included in this study. The HCs were volunteers recruited by an advertisement. The volunteers had no history of neurological disorders or psychiatric illnesses and no gross abnormalities on brain MR.

This study was approved by the local Ethics Committee of the Hospital of Fuzhou and was performed following the approved guidelines. All participants gave informed consent to participate in the investigation.

### Data acquisition

Imaging was performed using a 3.0 T Siemens Vision Scanner (Erlangen, Germany) equipped with high-speed gradient. The following parameters were used for 3D T1 imaging: repetition time/echo time (TR/TE) = 2300/2.98 ms, matrix = 512 × 512, flip angle = 9°, voxel size = 0.5 × 0.5 × 1 mm^3^, 176 axial slices without interslice gap. Functional images were acquired from the same locations as the anatomical slices using an echo-planar imaging sequence with the following parameters: TR/TE = 2000/30 ms, matrix = 64 × 64, flip angle = 90°, interslice gap = 4.0 mm, voxel size = 3.8 × 3.8 × 4 mm^3^, and slices = 31. For each participant, the fMRI scan lasted for 6 min, and 190 volumes were obtained.

### Lesion mapping

We constructed a lesion overlap image for all aphasic patients. A radiologist (Y.L.) manually traced the outline of the lesion on individual 3D T1 images using MRIcron (http://www.mccauslandcenter.sc.edu/mricro/mricron/), thereby creating a lesion mask for each patient. After the spatial normalization process, the union of all individual lesion masks was used to construct a group lesion mask for the patients (Fig. 1).

### Data preprocessing

Functional images were preprocessed using DPARSF (http://www.restfmri.net)^43^ and SPM8 (http://www.fil.ion.ucl.ac.uk/spm) toolkits. The first 10 functional volumes were discarded as signal equilibrium and adaptation to scanning noise by the subjects. We corrected the remaining images for temporal differences and head motion. No translation or rotation parameters in any given data set exceeded ±1 mm or ±1°. We also calculated individual mean frame-wise displacement (FD) by translation and rotation parameters of head motion based on the formula from a previous study^44^ and to evaluate group differences. No difference was observed for the mean FD between groups (Mann Whitney U-test, P = 0.19). Spatial normalization of the functional images was performed using 3D T1-based transformation. We coregistered individual 3D T1 images to functional images. The 3D T1 images were segmented and normalized to Montreal Neurologic Institute (MNI) space by a 12-parameter nonlinear transformation. In addition, we used a cost-function modification to exclude the lesion area, avoiding bias during spatial normalization^45^. This process has been implemented in SPM8 and adopted in other brain imaging studies with lesions^46^. These transformation parameters were applied to functional images. After spatial normalization, functional images were resampled at 3 × 3 × 3 mm^3^ voxel size. We spatially smoothed the images with an 8 mm full-width half-maximum isotropic Gaussian kernel. Finally, we removed linear trends from the time courses and with temporal band-pass filtering (0.01–0.08 Hz).

### Intrinsic regional activity analysis

We used ALFF to characterize the intrinsic regional activity at each voxel^47^. The time series for each voxel was transformed to the frequency domain using Fast Fourier Transform, and the power spectrum was then obtained. The power of a given frequency is proportional to the square of the amplitude of this frequency component. Thus, the square root was calculated at each frequency of the power spectrum, and the averaged square root was obtained across 0.01–0.08 Hz at each voxel. The averaged square root was considered as the ALFF. Each individual ALFF map was z-score standardized to allow further comparison between groups^48^. We created a patient specific group mask, such that, the gray matter template excluded the patients’ group lesion mask. The ALFF maps for the patient group were then standardized by subtracting the ALFF value in the patients’ group mask from the mean the value at each voxel and divided the value at each voxel by the standard deviation within the patients’ group mask. The ALFF maps for the HC group were also similarly standardized via the standard deviation within the gray matter template.

### Interregional functional connectivity analysis

In addition, interregional FC was analyzed. Group level brain regions that showed significantly altered ALFF in MNI space and regions that showed correlation with clinical scores for the ABC in aphasia patients were defined as seed regions for subsequent FC analysis. In this case, we would detect the functional integration map of the brain regions that showed altered regional brain activity using seed-based functional connectivity. The averaged time course was obtained from the seed region, and correlation analysis was performed using a voxel-wise technique to generate the FC map. In addition, six motion parameters, cerebrospinal fluid, and white matter signals were removed as nuisance variables to reduce the effects of head motion and non-neuronal fluctuations.

### Statistical analysis

Two-sample t-tests were performed on individual standardized ALFF maps by the SPM8 toolkit to investigate differences in intrinsic regional activity between aphasic patients and HCs. Group comparison was applied within the patients’ group masks to exclude the lesions in all patients. We included age, gender, and education level as covariates. The significance threshold was set to an FDR corrected p value <0.05 and minimum cluster size of 30 voxels. The minimum cluster size was chosen based on the AlphaSim program in the REST software (http://www.restfmri.net)^49^. This software applies Monte Carlo simulation to calculate the probability of false positive detection by considering individual voxel probability thresholding and cluster size. We computed this number of voxels by the estimated smoothness with a statistical map (two sample t-test map) under the patients’ group mask. The same procedure was applied for inter-regional FC between group comparisons. The automated anatomical labeling (AAL) atlas^50^ was used to identify the regions showing significant differences in the ALFF and FC analyses.

Finally, we used Pearson correlation to determine whether the abnormal ALFF regions were correlated with the clinical scores for the ABC in aphasic patients. We determined the mean z-value of each patient in the region of interest, which was the abnormal region in aphasic patients, according to the result of the two-sample t-test. We then computed the Pearson correlation coefficient among these ALFF values and the clinical scores for the ABC. Given that these analyses were exploratory, we used an uncorrected statistical significance level of p < 0.05.

## Additional Information

**How to cite this article**: Yang, M. *et al*. Altered Intrinsic Regional Activity and Interregional Functional Connectivity in Post-stroke Aphasia. *Sci. Rep*. **6**, 24803; doi: 10.1038/srep24803 (2016).

## Figures and Tables

**Table 1:** Demographic and Clinical Characteristics for Subjects.

Characteristics	Aphasia	HC	
	(n = 17)	(n = 20)	P value
Handedness (left/right)	0/17	0/20	–
Gender (M/F)	11/6	12/8	0.77^a^
Age (years)	53.53 ± 14.06	54.05 ± 8.43	0.89^b^
Education (years)	8.71 ± 1.26	8.45 ± 1.47	0.58^c^
Lesion size (cm^3^)			
Time post-stroke (days)	9.72 ± 5.30		
ABC scores		–	–
Aphasia Quotient (AQ)	40.88 ± 13.57	–	–
	97.11 ± 2.43*		
	93.25#		
Spontaneous speech score	8.82 ± 6.74	–	–
	19.65 ± 0.66*		
Auditory comprehension score	145.12 ± 47.16	–	–
	193.08 ± 7.96*		
Repetition score	87.53 ± 23.67	–	–
	96.64 ± 4.72*		
Naming score	36.94 ± 33.50	–	—
	95.84 ± 3.81*		
Performance Quotient (PQ)	22.20 ± 11.13	–	–
Reading/writing score	90.44 ± 60.91	–	–
Praxis score	44.76 ± 16.41	–	–
	59.91 ± 0.29*		
Construction score	56.68 ± 28.32	–	–
	86.04 ± 8.42*		
Cortical Quotient (CQ)	49.60 ± 19.55	–	–
	95.57 ± 3.01*		
	90.85#		

HC: healthy subjects; Data values are Mean ± SD.

^a^Chi-square test; bMann Whitney U-test.

^b^Two sample t-test.

^*^Normative scores (mean ± SD) for healthy controls (see ref. 38).

^#^Cut-off scores based on the receiver operating curve analyses (see ref. 38).

**Table 2:** Stroke-related clinical characteristics for patients.

no	Gender/ Age (years)	Educ. (years)	Aphasia type	Site of lesion	Size of lesion (cm^3^)	Time post-stroke (days)	AQ (0–100)#	PQ (0–40)#	CQ (0–100)#	SS (0–20)#	AC (0–200)#	R (0–100)#	N (0–100)#	R&W (0–200)#	P (0–60)#	C (0–100)#
1	M/51	10	Conduction	Frontal, limbic	119.22	14	40.6	10.1	35.7	4	106	100	48	17	43	12
2	F/71	10	Conduction	Frontal, parietal, insular	145.58	16	46.2	29.1	61.5	10	186	100	33	128	54	73
3	M/33	9	Broca’s	Temporal, occipital	101.62	5	25.4	3.1	19.9	4	82	76	8	9	6	12
4	M/44	8	anomic	Frontal, parietal	70.38	16	60.6	33.7	74.0	18	200	100	94	156	60	80.5
5	M/60	9	anomic	Temporal, occipital	159.56	11	34.7	6.0	27.9	6	91	86	39	14	18	16
6	F/47	9	anomic	Frontal, insular	177.86	2	42.3	33.5	64.2	13	190	100	10	142.5	60	92.5
7	M/63	8	anomic	Temporal, occipital	68.57	5	53.2	28.2	63.9	17	183	100	66	107.5	56	81
8	F/65	6	anomic	Temporal, occipital	125.14	9	50.2	35.4	70.2	17	193	100	46	167	60	87
9	F/77	10	Conduction	Subcortical, insular	75.80	7	32	18.2	39.7	2	110	92	12	72	40	43
10	M/54	7	anomic	Temporal	48.74	17	51.8	30.5	66.3	10	198	95	60	135.5	56	76.5
11	M/37	7	anomic	Temporal, occipital	46.86	3	61	35.0	75.5	18	200	100	96	177	60	72.5
12	M/69	9	anomic	Frontal, temporal	1.68	15	57.4	36.4	74.3	18	184	100	86	172.5	60	91.5
13	M/38	9	anomic	Frontal, temporal, insular	122.78	17	27.7	15.9	34.2	2	89	90	3	68.5	24	50.5
14	F/57	8	Conduction	Temporal	32.15	8	37.2	16.9	35.5	1	101	85	0	44.5	42	54
15	M/64	11	Conduction	Frontal, temporal, limbic	20.01	14	34.2	18.6	41.7	7	121	88	19	63.5	46	45.5
16	M/51	10	Conduction	Temporal, occipital	225.77	3	25	12.7	30.0	2	96	74	2	37	42	20
17	F/29	9	Broca’s	Temporal, limbic	76.55	7	15.4	13.9	28.4	1	137	2	6	26	34	56

M, male; F, female; PQ, Performance Quotient; CQ, Cortical Quotient; Educ., Education; SS, Spontaneous speech; AC, Auditory comprehension; R, Repetition; N, Naming; R&W, Reading&writing; P, Praxis; C, Construction.

^#^indicates the ranges for each subtest.

**Table 3:** Regions showing abnormal amplitude of low-frequency fluctuation in patients.

Brain regions	Brodmann area	MNI (x, y, z)	Cluster size(voxels)	T value
Aphasia > HC				
R MTG	21	(72,−18,−15)	282	7.08
R TPOmid	21	(60, 12 −24)	69	5.73
R ITG	20	(48, −36, −24)	206	5.61
R FFG	20	(42, −18, −36)	73	5.06
R HIP/ParaHip	28	(33, −21, −12)	60	4.91
Aphasia < HC				
L/R SMA	6	(−3, 15, 72)	491	−10.31
L SFG	6	(−12, −9, 81)	53	−6.63
L/R LING	18	(−3, −78, −6)	106	−6.43
L PreCG	6	(−18, −21, 81)	47	−5.82
R PreCG	6	(18, −18, 81)	43	−5.19
L/R ACC	32	(−3, 33, 30)	38	−4.63

Abbreviations: ACC, anterior cingulate cortex; FFG, fusiform gyrus; ParaHip, parahippocampus; HIP, hippocampus; ITG, inferior temporal gyrus; LING, lingual gyrus; MTG, middle temporal gyrus; PreCG, precentral gyrus; SFG, superior frontal gyrus; SMA, supplementary motor area; TPOmid, middle temporal pole. x, y, z, coordinates of primary peak locations in the Montreal Neurological Institute (MNI) space; T value, statistical value of peak voxel showing ALFF differences between the groups.

**Table 4:** Seed-based functional connectivity abnormalities in aphasia patients.

Seed region	Connective regions	Brodmann area	MNI (x, y, z)	Cluster size (voxels)	Connectivity strength (r value)		Connectivity difference (T value)
					HC	Aphasia	Aphasia vs. HC
	R FFG	37	(33, −48, −18)	33	0.30 ± 0.17	0.45 ± 0.20	3.96
	L MOG	19	(−27, −87, 36)	370	0.27 ± 0.10	0.05 ± 0.19	−5.89
R Hip/ParaHip	L TPOmid	36, 38	(−24, 12, −33)	63	0.35 ± 0.14	0.08 ± 0.22	−4.50
	L PCL	6	(−9, −21, 81)	60	0.22 ± 0.18	−0.08 ± 0.20	−4.74
	L PostCG	6	(−21, −30, 78)	143	0.19 ± 0.17	−0.04 ± 0.15	−4.25
	L TPOsup	38	(−39, 27, −30)	32	0.31 ± 0.15	0.05 ± 0.19	−3.95

Abbreviations: FFG, fusiform gyrus; MOG, middle occipital gyrus; PCL, paracentral lobule; PostCG, postcentral gyrus; TPOmid, middle temporal pole; TPOsup, superior temporal pole; x, y, z, coordinates of primary peak locations in the Montreal Neurological Institute (MNI) space; T value, statistical value of peak voxel showing FC differences between the groups.
